# CIRCULATING IMMUNE CELLS ARE ASSOCIATED WITH BRAIN MRI MEASUREMENTS IN THE FRAMINGHAM OFFSPRING

**DOI:** 10.1093/geroni/igad104.3226

**Published:** 2023-12-21

**Authors:** Yumeng Cao, Margaret Doyle, Jiachen Chen, Ahmed A Y Ragab, Joanne Murabito, Kathryn Lunetta

**Affiliations:** Boston University, Boston, Massachusetts, United States; University of Vermont, Colchester, Vermont, United States; Boston University School of Public Health, Boston, Massachusetts, United States; Boston University School of Public Health, Boston, Massachusetts, United States; Boston University School of Public Health, Boston, Massachusetts, United States; Boston University School of Public Health, Boston, Massachusetts, United States

## Abstract

Mounting evidence supports the role of the immune system in brain health, yet little is known about the role of circulating immune cells in brain health. We investigated the association of immune cell phenotypes with brain MRI phenotypes in the Framingham Heart Study Offspring participants who attended Exam 7 (mean age 61, 52% women). Immune cells (43 analyzed here) were determined by flow cytometry and previously published. Analysis of the immune cell phenotypes with MRI measurements of 13 brain regions revealed several significant associations, adjusting for age, sex and CMV status (Model 1) in the full sample (n=703) and the CMV+ stratum (n=327). In the full sample, higher senescent/exhausted CD8+ cells (CD8+CD45RO-CCR7-CD27-(Teff), CD8+CD45RA+CD28-CD57+(TEMRA), CD8+CD27-CD28-) associated with higher cerebrum and frontal gray volumes. These associations were strengthened when cardiovascular risk factors were included in the model (Model 2). In the CMV+ stratum, two CD8+ cell types (TEMRA, and Teff) were significantly associated with higher cerebrum gray and frontal gray matter volumes in both models. Also in the CMV+ stratum, higher levels of CD8+CD45RO+CCR7+CD27+ (Central Memory) were associated with lower frontal gray matter, and higher CD3+ (T cells) cells were associated with higher temporal gray matter and higher CD8 Teff associated with lower cerebrum white volumes. There were no associations of CD4 subtypes with brain MRI measures in the full sample or the CMV+ stratum. These associations with brain volumes in adults aged 40-88 may improve understanding of the role of the immune system early in the brain aging process.

